# Cations Do Not Alter the Membrane Structure of POPC—A Lipid With an Intermediate Area

**DOI:** 10.3389/fmolb.2022.926591

**Published:** 2022-07-11

**Authors:** Sergei Kurakin, Oleksandr Ivankov, Vadim Skoi, Alexander Kuklin, Daniela Uhríková, Norbert Kučerka

**Affiliations:** ^1^ Frank Laboratory of Neutron Physics, Joint Institute for Nuclear Research, Dubna, Russia; ^2^ Institute of Physics, Kazan Federal University, Kazan, Russia; ^3^ Moscow Institute of Physics and Technology, Dolgoprudnyi, Russia; ^4^ Department of Physical Chemistry of Drugs, Faculty of Pharmacy, Comenius University Bratislava, Bratislava, Slovakia

**Keywords:** lipid bilayer, structure, cations, SAXS, SANS, densitometry

## Abstract

Combining small-angle neutron scattering (SANS), small-angle X-ray scattering (SAXS), and densitometric measurements, we have studied the interactions of the divalent cations Ca^2+^ and Mg^2+^ with the lipid vesicles prepared of a mixed-chain palmitoyl-oleoyl-phosphatidylcholine (POPC) at 25°C. The structural parameters of the POPC bilayer, such as the bilayer thickness, lateral area, and volume per lipid, displayed no changes upon the ion addition at concentrations up to 30 mM and minor changes at > 30 mM Ca^2+^ and Mg^2+^, while some decrease in the vesicle radius was observed over the entire concentration range studied. This examination allows us to validate the concept of lipid–ion interactions governed by the area per lipid suggested previously and to propose the mixed mode of those interactions that emerge in the POPC vesicles. We speculate that the average area per POPC lipid that corresponds to the cutoff length of lipid–ion interactions generates an equal but opposite impact on ion bridges and separate lipid–ion pairs. As a result of the dynamic equilibrium, the overall structural properties of bilayers are not affected. As the molecular mechanism proposed is affected by the structural properties of a particular lipid, it might help us to understand the fundamentals of processes occurring in complex multicomponent membrane systems.

## Introduction

The lipid composition is extremely diverse within both the cell membrane and the organelles that make up the cell. The ratio of lipids, differing in the chain length and saturation, charge, and size of the head groups, varies among different organelles, and it is related to their certain function in a body. Some of the most important and biologically relevant lipids are unsaturated lipids, which are widespread not only in cell membranes throughout an organism but also in the synaptic vesicles, the endoplasmic reticulum (ER), or Golgi apparatus of eukaryotic cells (Schneiter et al., 1999; van Meer et al., 2008; Antonny et al., 2015). Among all lipid species, POPC is an example of mixed saturated/unsaturated-chain lipid that is highly abundant in the lipid bilayers of different cell constituents (Petrache, 2012).

All of these cellular components are found in the medium, which is an electrolyte solution that contains a huge variety of ions. Among all ions, divalent cations of alkaline Earth metals, such as Ca^2+^ and Mg^2+^, appear to be of specific interest because of their vast number of features and their contribution to many cellular processes. They are necessary for cell activity at certain concentrations and take part directly in the mineralization and contraction of tissues, signal transduction, protein synthesis, and regulation of protein–lipid interactions (Beto, 2015; Gröber et al., 2015). While the concentration of Ca^2+^ varies greatly and it is relatively small in the cytoplasm and intracellular medium of different organelles and tissues, varying even from a nanomolar to micromolar range in ER (Xu et al., 2014) and reaching 1–2 mM values outside of cells (Bagur and Hajnóczky, 2017), the concentration of Mg^2+^ is often in the millimolar range in mammalian cells and even reaches 15 mM for ER (Romani and Scarpa, 1992; Romani, 2011). In addition, in the cells, the rapid ion spikes caused by membrane influxes of, for example, Ca^2+^ into the cytosol via ion channels are able to increase the Ca^2+^ concentration locally by dozens of times (Berridge et al., 2003). All these concentrations in the cell environment are basically maintained by concentration gradients employing the binding of ions to phospholipid head groups, membrane-embedded proteins, peptides, and other different membrane components. This binding leads to the changes in membrane structures, mechanisms of interactions between membrane constituents, and effects on the functions and conformations of various proteins and peptides integrated into it (Lee, 2004), sometimes resulting in devastating aftermaths. For example, it has been suggested that the onset of conformational diseases, such as Alzheimer’s disease, is related to the mechanisms based on the violations of neuronal calcium homeostasis, especially the interactions between Ca^2+^ cations and amyloid β-peptides intercalated into membranes (Mattson et al., 1992).

The changes in the structure of lipid membranes under the influence of ions have been thoroughly studied by diverse research methods and experimental techniques, such as X-ray and neutron methods of scattering and diffraction, infrared and NMR spectroscopy, calorimetry, molecular dynamics (MD) simulations, and others (Lindblom and Lindman, 1973; Lis et al., 1981; Altenbach and Seelig, 1984; Lehrmann and Seelig, 1994; Binder and Zschornig, 2002; Petrache et al., 2006a; Petrache et al., 2006b; Sinn et al., 2006; Pabst et al., 2007; Gorshkova et al., 2017; Javanainen et al., 2017; Kučerka et al., 2017b; De Mel et al., 2020; Javanainen et al., 2020). When using these methods, it has been reported about the structural organization of membranes, namely, the bilayer thickness and lateral area usually affected by the conformational changes of the head groups (Seelig, 1990; Melcrová et al., 2016), ordering of acyl chains (Huster et al., 1999; Kučerka et al., 2021), and lipid hydration and dehydration (Martín-Molina et al., 2012; Kanti De et al., 2018) inside lipid bilayers upon ion additions. Some individual peculiarities of ions, namely, their localization and distribution near lipid head groups and effect of ion hydration on the binding effect to membranes, have also been revealed (Tsai et al., 2012; Alsop et al., 2016; Le et al., 2019; Kučerka et al., 2021). In addition, the impact of lipid charge (zwitterionic vs. anionic lipids) on the ion binding and membrane structure has been shown. Unlike zwitterionic membranes, a lipid bilayer composed of the zwitterionic/anionic lipid mixture is subjected to the overcharging effects upon ion binding, as well as different tilting of lipid head groups and slightly peculiar ion localization at the membrane–water interface, as discovered recently by MD simulations and various experimental techniques (Melcrová et al., 2016; Valentine et al., 2020).

Interactions of different divalent cations with lipid membranes are nontrivial and cause certain challenges in their study. In MD simulations, divalent cations pose some problems to traditional non-polarizable force fields. A thorough work for reducing the ion overbinding through the comparison of force fields in the case of PC and PS lipids (Catte et al., 2016; Botan et al., 2015) was accomplished within the framework of NMRlipids project (http://nmrlipids.blogspot.com). On the other hand, being measured experimentally, calcium and magnesium ions are known to affect the bilayer thickness, water layer between two adjacent bilayers, and area per lipid (*A*_*L*_) differently to some degree. Magnesium ions lead to the monotonous bilayer repulsion as a function of their concentration (Alsop et al., 2016), whereas calcium ions demonstrate the attraction of bilayers at a certain concentration and, moreover, all these cations induce a similar but non-linear effect on the bilayer itself (thickness, *A*_*L*_, and lipid dehydration) (Uhríková et al., 2008; Kurakin et al., 2021). However, our latest study (Kučerka et al., 2021) revealed some general patterns of calcium and magnesium impact on the structural parameters of lipid bilayers in unilamellar vesicles (ULVs) that allows us to define two types of basic interactions of ions and phospholipids, whose packing density in the lipid bilayer differs. Based on the observations of changes in the bilayer thickness and consequently *A*_*L*_, this manifests the essential role of *A*_*L*_ determined by, for example, the saturation of acyl chains, which allows the lipid–ion interactions to be divided into two binding modes. Saturated phospholipids, such as dipalmitoylphosphatidylcholine (DPPC) or dimiristoylphosphatidylcholine (DMPC), have a “small” *A*_*L*_ (< 54 Å^2^ in gel phase or < 63 Å^2^ in fluid phase) (Tristram-Nagle et al., 2002; Kučerka et al., 2011; Kučerka et al., 2021) that allows a preferential lipid–ion–lipid bridging, while di-monounsaturated dioleoylphosphatidylcholine (DOPC) having a “large” *A*_*L*_ (> 67 Å^2^) (Kučerka et al., 2008) forms separated lipid–ion pairs.

In this work, we extend our previous studies of lipid–ion interactions and concentrate our efforts on the experimental research of the mixed-chain POPC bilayer. Intriguingly, its lateral area equals ∼64 Å^2^ (at 25°C) (Kučerka et al., 2011) that appears to correspond to the cutoff length of the lipid–ion bridge (Ronnest et al., 2016; Javanainen et al., 2020). To this end, all our samples were prepared in the form of vesicles dispersed in the excess water. The combination of densitometry, SANS, and SAXS has made it possible to directly determine how *A*_*L*_ correlates to cation concentrations at the millimolar scale.

## Materials and Methods

### Materials

A highly purified (> 99%) 1-palmitoyl-2-oleoyl-*sn*-glycero-3-phosphocholine (POPC) lipid powder was purchased from Avanti Polar Lipids (Alabaster, United States) and used without further purification. The chloroform/methanol solvents and salts of CaCl_2_⋅2H_2_O/MgCl_2_⋅6H_2_O (Sigma-Aldrich, Germany) were also over 99% pure and used as received. Ultrapure H_2_O (18.2 MΩcm at 25°C) was obtained from the MilliQ purification system.

### ULV Preparation for SANS and SAXS Measurements

The method of lipid film hydration was applied for the preparation of multilamellar vesicles (MLVs). Samples were prepared at three different lipid concentrations: 0.5, 1, and 3 wt% by depositing to the plastic containers ∼ 6 mg (0.5 wt%), 12 mg (1 wt%), or 24 mg (3 wt%) of lipid powder dissolved in a mixture of chloroform (CHCl_3_) and methanol (CH_3_OH) solvents taken in a volume ratio of 2:1. The solvents were evaporated under a flow of nitrogen to create a lipid film at the bottom of the vials. The complete removal of solvent traces in the samples was achieved in a vacuum chamber by pumping out the air for approximately 12 h. Salts of CaCl_2_⋅2H_2_O and MgCl_2_⋅6H_2_O were used to prepare water stock solutions in the ion concentration range of 0–50 mM (in H_2_O for SAXS and D_2_O for SANS measurements), which were then used to hydrate the lipids in the containers for achieving the desired salt concentration. The system was thoroughly mixed in a shaker and subjected to freeze–thaw cycles (10 times) in order to obtain the MLV solution with a uniform distribution of all components inside. The point of using D_2_O is to maximize the contrast between the hydrogenated membrane and deuterated solvent to take full advantage of the SANS experimental approach.

ULVs were obtained by extruding the solution of multilamellar vesicles. We used two polycarbonate filters with a pore diameter of 500 Å (Avanti Polar Lipids, Alabaster, United States) in an Avanti Polar Lipids extruder (Alabaster, United States) equipped with two gastight syringes from Hamilton (Reno, Nevada, United States). The extrusion process was mechanically performed an odd number of times to prevent large vesicles from entering the sample. The samples were subjected to 31 passes through the filter at a room temperature that is significantly above the main phase transition temperature of pure POPC (*T* = −3°C) (Koynova and Caffrey, 1998). The resulting solution displayed a slight opalescence, which is typical for the dispersion of ULVs. After extrusion, the unilamellar vesicle solutions were sealed and incubated for at least 24 h at room temperature to achieve equilibrium in this system. All samples were extruded maximum 1–3 days before the experiment to avoid the spontaneous formation of multilamellar vesicles due to their possible fusion. The samples were finally placed in 1 mm light path quartz Hellma cells (Müllheim, Germany) or 1.5 mm quartz capillaries (Hilgenberg GmbH) for SANS and SAXS measurements, respectively.

### MLV Preparation for Densitometric Measurements

Dry lipid was weighed into plastic containers and hydrated in the freshly prepared MilliQ H_2_O water containing Ca^2+^ or Mg^2+^ cations in the desired concentration added from a stock solution. The final concentration of lipids for density measurements was 1 wt%. The system was mixed in a shaker and homogenized by the freeze–thaw cycles resulting in the MLV solution. To prevent the bubble formation, which often occurs during the prolonged density measurements and sample heating in the tube of a densitometer (Murugova and Balgavý, 2014), all the samples were degassed under low pressure by stirring the solution for 15–20 min and measured immediately without shaking and disturbing after this manipulation.

It should be emphasized that the densitometry experiments were not carried out on the ULV to avoid any possible changes in the concentration of ions and lipids due to their accumulation on filter pores during the extrusion procedures.

### SANS and SAXS Experiments

All neutron experiments were performed on a YuMO time-of-flight small-angle neutron scattering instrument, located at the fourth neutron guide of the IBR-2 pulsed nuclear reactor (Frank Laboratory of Neutron Physics, JINR, Dubna, Russia) serving as the source of neutrons (Kuklin et al., 2018). A beam of cold neutrons with a cold-moderator setup (Anan’ev et al., 2014) was focused by a set of two collimators with diameters of 40 and 14 mm. The neutrons scattered from the sample were recorded by two ring detectors located at distances of 4.5 and 13 m from the sample position, which made it possible to cover the range of the scattering vector *q* from 0.005 to 0.5 Å^−1^ (*q = (*4*π/λ)*sin*(θ/*2), where *λ* is the wavelength and *θ* is the scattering angle). The cold neutrons provide better data resolution at a low *q*-range, which allows resolving the shape of studied objects and their changes upon the addition of ions.

The vanadium standard scatterer was used to calibrate the absolute coherent scattering intensity, and the buffer solution was used to calibrate the background intensity. The temperature of all the samples was set to 25°C to study the fluid phase of the POPC lipid systems, and it was controlled electronically with an accuracy of ± 0.03°C using a Lauda thermostat with a Pt-100 temperature probe. The acquisition time for one sample was 15 min.

The SANS intensities were processed using the SAS software package providing the statistically treated data points with standard deviations (Soloviev et al., 2017). The resulting scattering curves were analyzed by SasView 4.2.2 software (SasView, 2022), utilizing the models available therein and are described in the Supplementary Material. The uncertainties of fitted parameters were calculated from the covariance matrix multiplied by the root of normalized chi-square.

SAXS experiments were performed using a Rigaku instrument at the Moscow Institute of Physics and Technology (Dolgoprudny, Russia). The Rigaku instrument has a pinhole camera, which is attached to a rotating anode of the X-ray high-flux beam generator (MicroMax 007-HF) providing 1200 W at the voltage and current of 40 kV and 30 mA, respectively (Murugova et al., 2015). The multiwire, gas-filled area detector with an active area of 20 cm diameter is built into the pinhole camera. The two-detector geometry was also applied in this case to cover the broad *q*-range (0.004–1.3 Å^–1^).

The SAXS curves of scattering intensities with their standard deviations as functions of the scattering vector amplitude were obtained using the SaxsGui 2.15.01 software package and analyzed by the SASfit 0.94.11 package (Bressler et al., 2015), as described in the Supplementary Material. The uncertainties of fitted parameters were calculated from the covariance matrix multiplied by the root of normalized chi-square.

### Densitometry

Density measurements were performed using the DMA 5000 M densitometer from Anton Paar (Graz, Austria). The basics of vibrational densitometry described elsewhere (Kratky et al., 1969) implies the measuring of the oscillation frequency of an internal U-shaped tube filled with the studied solution to determine its density. The instrument achieves a temperature accuracy of 0.01°C and that of the measured density of 0.000007 g/cm^3^ (https://www.anton-paar.com/corp-en/). The densitometer calibration was carried out by the measurements of air and water densities at a certain temperature. Between two adjacent measurements, the densitometer was thoroughly rinsed and dried.

## Results

Here, we report on our results of the density measurements of multilamellar vesicles. According to many studies, it is very important to take into consideration the lipid sedimentation and flotation processes that appear due to the difference in the density of lipids and the buffer during sample measurements, which in turn affects the accuracy of the molecular volume results (Hallinen et al., 2012; Murugova and Balgavý, 2014). Similar behavior of lipid dispersion has been described previously for zwitterionic DOPC (Murugova and Balgavý, 2014) and DPPC (Jones et al., 2012) lipids. To reduce the manifestation of this problem, we carried out all our density measurements in H_2_O instead of D_2_O buffer, as its density is closer to that of lipid. This alleviates lipid sedimentation. Also, we tested the changes in the density induced by sedimentation over the period of 8 h that corroborated the variation at a reproducibility level of 0.000005 g/cm^3^. Finally, we have measured the same samples several times and received data reproducibility characteristic to the utilized densitometer.

Temperature density measurements were performed at three different temperatures (20°C, 25°C, and 30°C). It is known that the volume per lipid was reported to vary with temperature, pressure, or when other molecules are added (Schmidt and Knoll, 1985; Skanes et al., 2006; Gallová et al., 2017), reflecting the changes in the inner structure and dynamics of lipid molecules. In our case, several samples were heated starting from 20°C to 30°C at the atmospheric pressure and then cooled to 20°C again, with no evidence of significant changes in the densities at the same temperatures. This indicates the absence of air bubbles that would potentially emerge in a significant amount during the heating, even though the temperature measurement was in a fairly narrow temperature range. This also confirms stable equilibrium conditions in which both types of our samples (with and without ions) are found.

**FIGURE 1:**
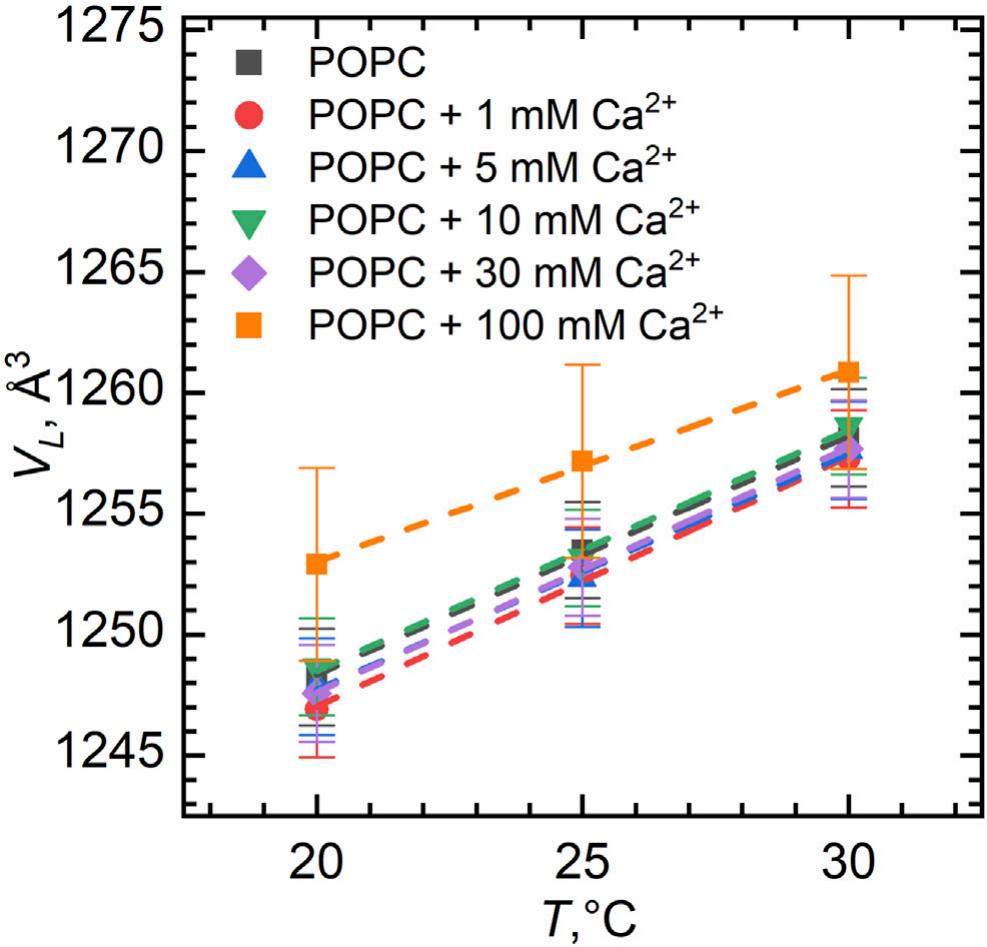
Volume per lipid *V*_*L*_ in POPC vesicles for different Ca^2+^ concentrations as a function of temperature. The error bars show systematic errors calculated based on sample preparation protocol (uncertainties in lipid weighing, and buffer volume added to the lipid powder, etc.).

Figure 1 represents the volume per lipid (*V*_*L*_) obtained according to Eq. 12 in Supplementary Material for different temperatures in an ion concentration range of 0–100 mM. We note a potential problem in this method of V_L_ determination related to the incorrect subtraction of the buffer density that corresponds to the alleged density of buffer in the given lipid dispersion while not assuming the depletion of ions in solution due to their adsorption on the bilayer. Nonetheless, the results presented (*V*_*L*_ = 1253 Å^3^ for POPC at 25°C in H_2_O) are very close to the previously published results extracted from the neutral buoyancy and densitometric measurements (Koenig and Gawrisch, 2005; Gironi et al., 2019). We estimate the experimental errors at a level of 2–4 Å^3^ based on the inaccuracies during preparing the samples and implementing instrumental measurements and slight discrepancies observed during heating/cooling cycles, as described above. This estimate coincides with the literature (Nagle et al., 2019).

We have observed a predictable increase in the volume per lipid upon its heating (Figure 1). This increase of *V*_*L*_ can be approximated by a linear function providing us with the coefficient of thermal volume expansivity at the constant pressure:

$$\beta =\frac{1}{V}\left(\frac{\partial V}{\partial T}\right)=\frac{\mathrm{\partial ln}V}{\partial T}.$$

The value of the coefficient *β* can be calculated from the slope of ln*V*_*L*_ vs. *T*. In our case, *β* = 79∗10^−5^ K^−1^ ± 3∗10^−5^ K^−1^ for pure POPC, which is found to be in a good agreement with the results published previously (Koenig and Gawrisch, 2005). Moreover, the coefficient of thermal volume expansivity little depends on the ion additions to the sample over the entire concentration range, as also seen from the slopes of lines assigned to different calcium concentrations.

**FIGURE 2:**
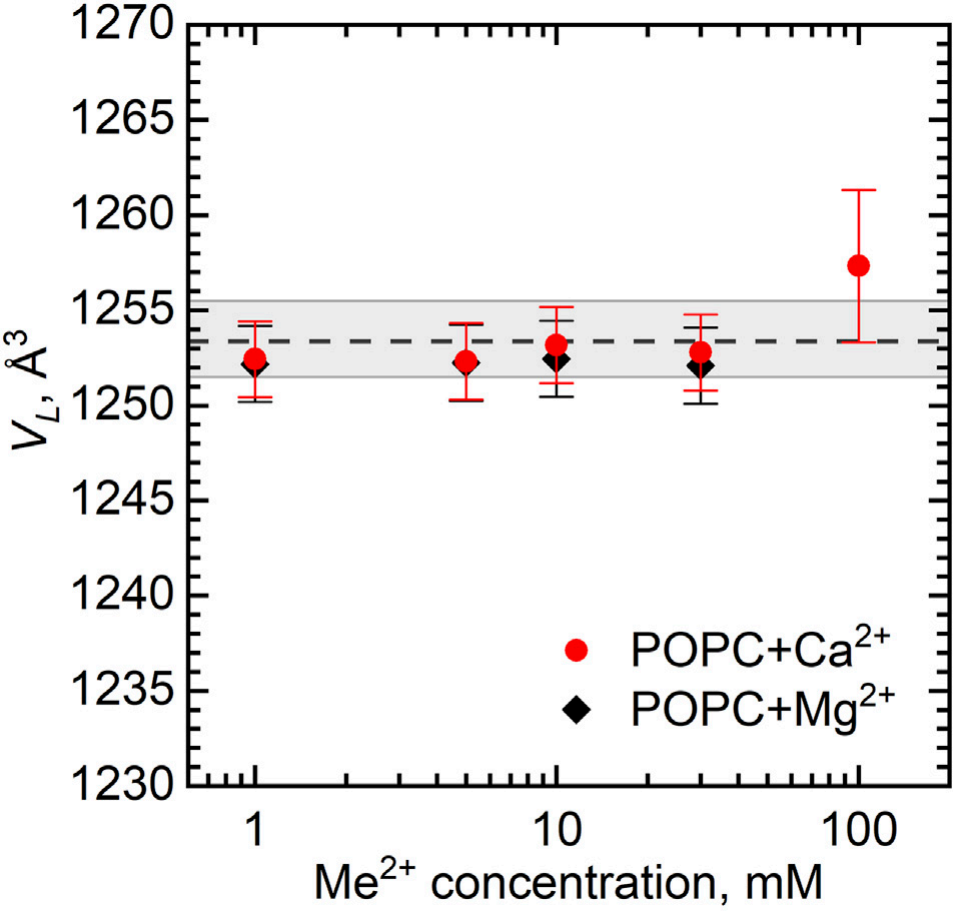
Dependence of volume per lipid *V*_*L*_ on the concentration of Ca^2+^ and Mg^2+^ in POPC vesicles at *T* = 25°C. The dashed line represents *V*_*L*_ of neat POPC MLVs with the wide gray band depicting its standard deviation.

Figure 2 shows the dependence of *V*_*L*_ as a function of Ca^2+^ and Mg^2+^ concentrations. The largest ion concentration studied appears to result in some variation in *V*_*L*_. It possibly relates to the changes in the hydration conditions (ions may dehydrate the lipid head groups) or it can be an artifact associated with excess subtraction of the buffer density (the concentration of ions in solution decreases as some ions bind preferentially to the lipid bilayer). The *V*_*L*_ changes over the rest of concentration range being well within the experimental uncertainties, however, prompt us to disregard the changes.

**FIGURE 3:**
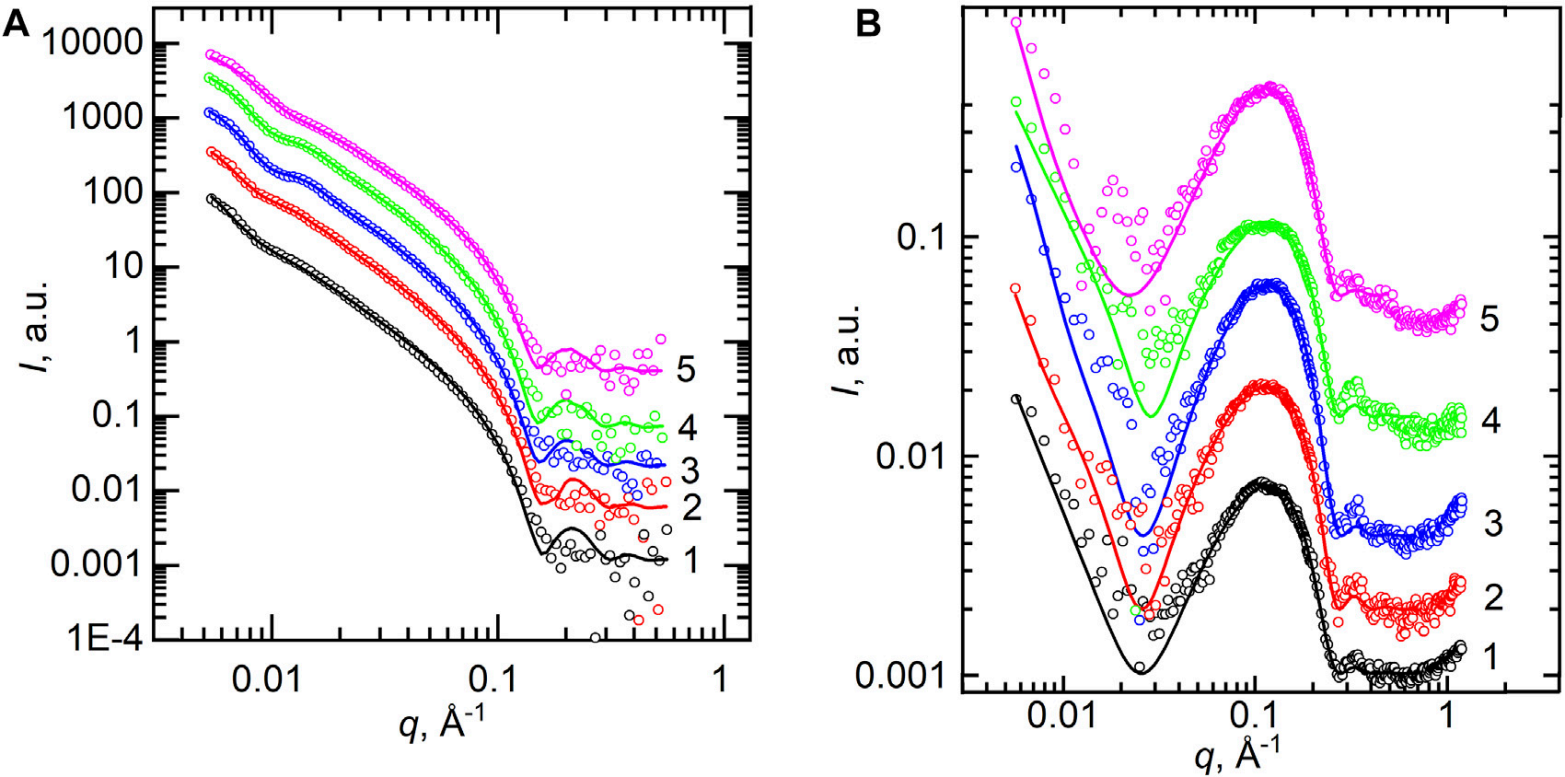
**(A)** SANS experimental curves measured at *T* = 25°C for POPC samples (points) and the best-fit results of their model analysis by spherical vesicles (lines). **(B)** SAXS curves measured at *T* = 25°C for POPC (points) and their best-fit results of the Gaussian model (lines). SANS and SAXS curves are presented for 0 mM (1), 1 mM (2), 5 mM (3), 10 mM (4), and 50 mM (5) of Ca^2+^ concentration.

Furthermore, we describe the structural results of POPC lipid bilayers obtained from SANS and SAXS techniques (Figure 3A and Figure 3B, respectively). The curves represent the scattered intensity *I(q)* vs. *q* for samples of different concentrations of calcium cations, where the points represent the experimental data and lines show the best fits. All the curves shown are typical for ULVs without any presence of the Bragg peak at *q* ∼ 0.1 Å^−1^ that would describe the multilamellar structures. However, a noticeable feature in the SANS curves is a systematic displacement of the pit visible around *q* ≈ 0.01 Å^−1^ to higher *q* values. This indicates a change in the vesicle sizes. *q* ≈ 0.01 Å^−1^ represents the reciprocal space length that corresponds to *d* = 2*π*/*q* ≈ 600 Å, which is a predicted diameter of our ULVs (Kučerka et al., 2007).

**FIGURE 4:**
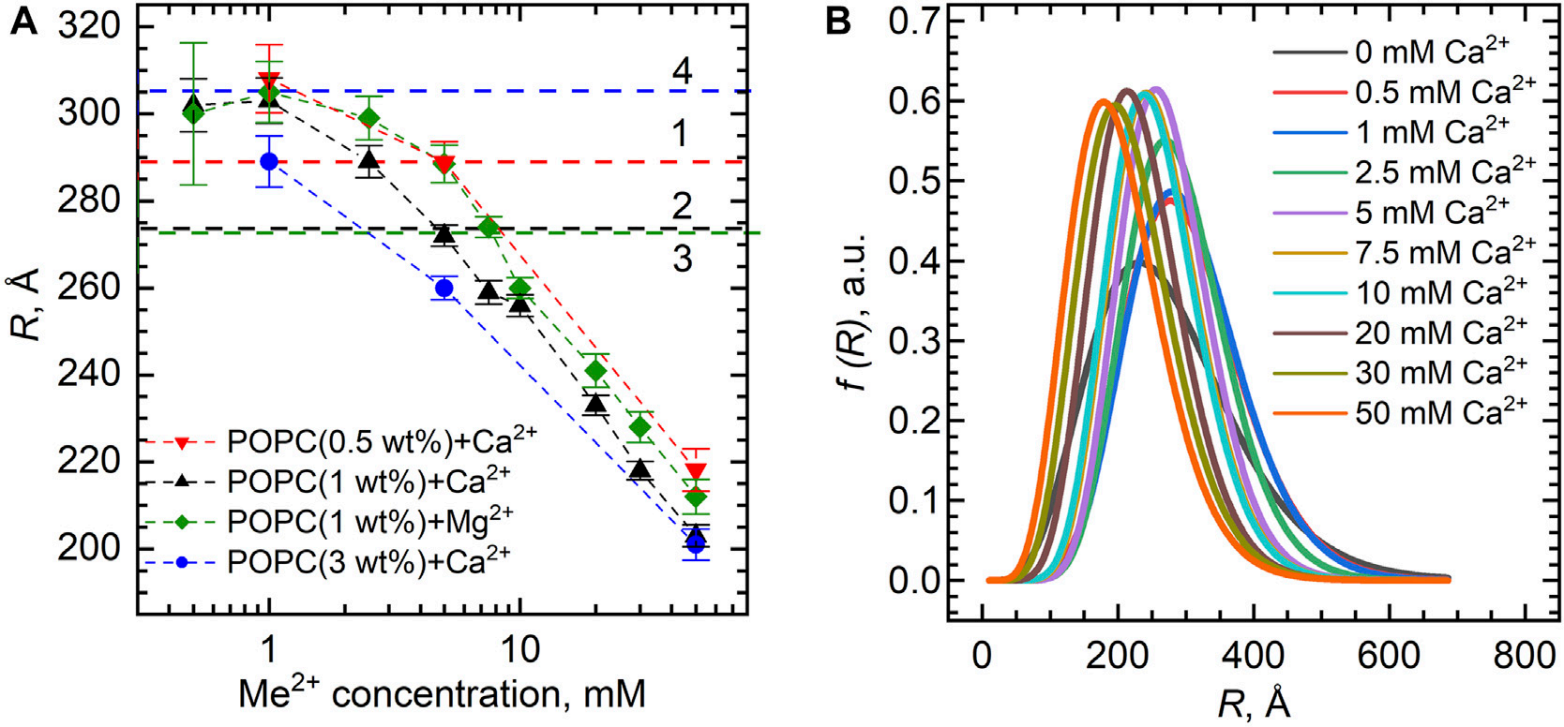
**(A)** Inner vesicle radii *R* extracted from the SANS experiments for the POPC bilayers (lipid concentrations of 0.5 wt%, 1.0 wt%, and 3.0 wt%) loaded with various Ca^2+^ and Mg^2+^ concentrations. Horizontal dashed lines represent *R* of POPC ULVs without Ca^2+^/Mg^2+^ obtained with the same model, where numbers (1–4) represent different POPC concentrations in the sample: 0.5 wt% (1), 1 wt% (2–3), and 3 wt% (4). **(B)** Distribution functions of POPC vesicle radii upon Ca^2+^ additions extracted from the fitting of SANS data. The functions are presented in accordance with the Schultz distribution described in the Supplementary Material.

Figure 4A shows the dependence of the vesicle inner radius *R* obtained from fitting the SANS data. For standardly prepared 1 wt% samples with either Ca^2+^ or Mg^2+^, we observed unexpectedly a distinct decrease in the average radius of the vesicles from about 300 Å down to about 200 Å at the 0–50 mM concentration range. This could be possibly a result of intervesicular interactions whose characteristic length would be affected by the concentration of vesicles. We have therefore examined two additional samples prepared at the lipid concentrations of 0.5 and 3 wt%. Our results, however, clearly invalidate this to be a cause of the observation.

Despite the obvious systematic changes, it is important to note the low-resolution ability of our data with respect to vesicle sizes, as documented by the characteristic pit at the low-*q* region of data being weakly pronounced. Using the fitting results, we plotted the distribution functions of vesicle radii at different ion concentrations based on the Schulz distribution (Figure 4B). Ignoring a small variation, the full width at half-maximum gives the size polydispersity of approximately 200 Å. This makes us deem our observation of vesicle size decrease a possible consequence of high polydispersities of the samples.

Our SAXS data contain even less reliable information about the size of vesicles due to poorer intensity resolution at a low *q*-range. On the other hand, acceptable statistics at high *q* values (*q* > 0.1 Å^−1^) provide more reliable information about the bilayer thickness. It is worth noting that both SANS and SAXS data allow us to focus on the overall bilayer thickness, while they complement each other with the vesicle parameters provided by SANS and additional details on the bilayer inner structure provided by SAXS (Kučerka et al., 2010). Thus, while we have fitted the SANS data with a model of spherical vesicles with a single-shell bilayer thickness *d*_*L*_, the SAXS data were fitted with the 3-Gaussian model providing *d*_*HH*_ thickness (i.e., the phosphorus–phosphorus distance defined as a distance between two head groups Gauss functions).

Scattering length density profiles are represented in Supplementary Figures S2, S3. In the model of spherical vesicles (Supplementary Figure S2), the nuclear distribution is presented as two different areas characteristic of the abrupt contrast between a H-rich lipid bilayer and D_2_O outside the bilayer. The electron density profiles depict lipid head groups via two peaks (Supplementary Figure S3)*.* We emphasize that the SAXS fitting procedure was performed with several fixed parameters (*σ*_*head*_, *σ*_*tail*_ *, R*, and *σ*_*R*_) and free parameters (*ρ*_*head*_ *, ρ*_*tail*_, and positions of the lipid head groups). Due to the fairly scarce resolution in a low *q*-range, fitting is rather insensitive to changes of parameters *R* and *σ*_*R*_ describing the vesicle radius and polydispersity. In addition, the fitting divergence was avoided by *σ*_*head*_ and *σ*_*tail*_ being strictly fixed to the values obtained previously (*σ*_*head*_ = 3.6 Å, *σ*_*tail*_ = 5.8 Å at *T* = 25°C) (Pabst et al., 2000).

**FIGURE 5:**
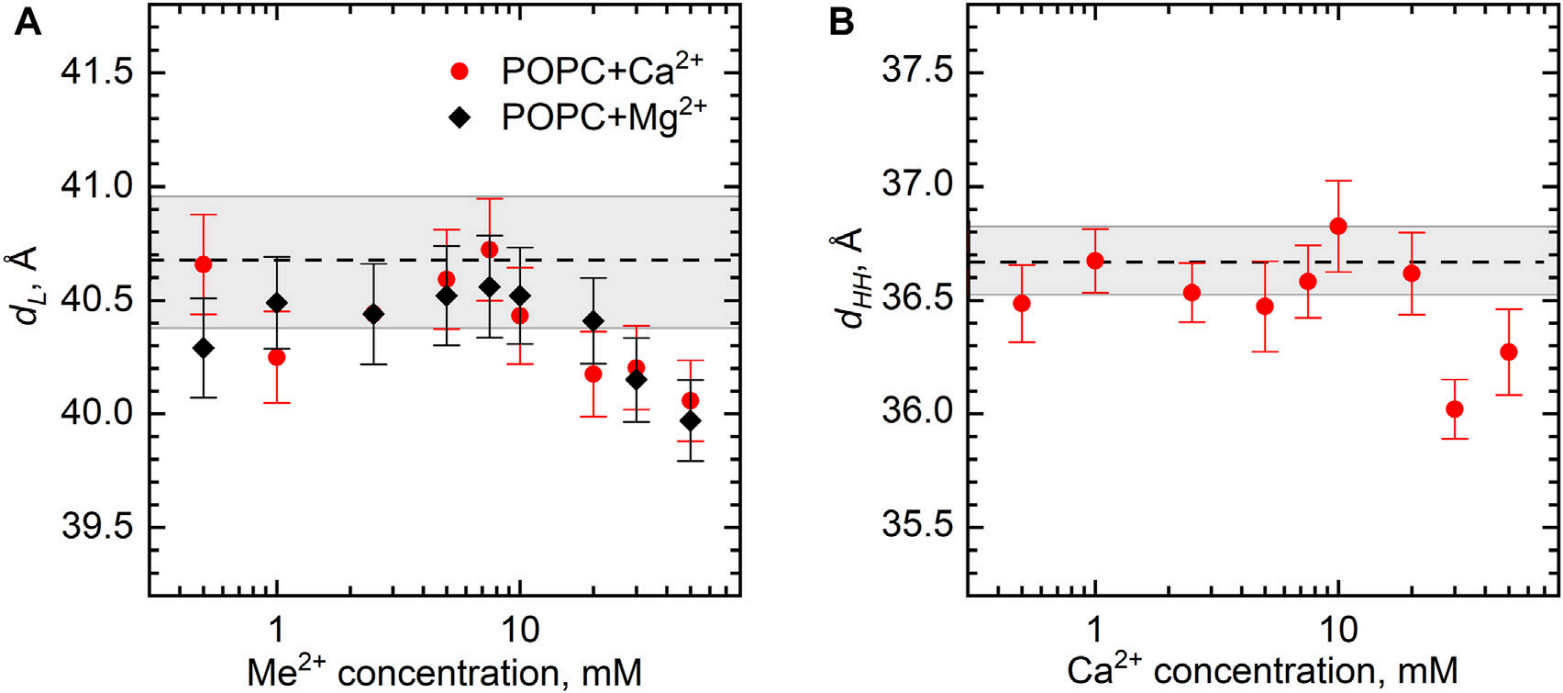
The changes of POPC bilayer thickness (*d*_*L*_ and *d*_*HH*_) as a function of Ca^2+^ and Mg^2+^ concentrations at 25°C according to **(A)** SANS and **(B)** SAXS measurements. Dashed lines represent *d*_*L*_ and *d*_*HH*_ of neat POPC ULVs with the wide gray bands depicting their standard deviations.

The structural parameters of the POPC bilayer itself, the bilayer thickness *d*_*L*_, and *d*_*HH*_, in particular, demonstrate the fairly constant value upon the addition of various amounts of Ca^2+^ and Mg^2+^ ions. The *d*_*L*_ gradually decreases from its original value of 40.5 Å by approximately 0.5 Å over a range of salt concentrations up to 50 mM (Figure 5A). Noteworthy is a similar result obtained for the samples with the lipid concentrations of 0.5 and 3 wt% (not shown). Finally, the same result follows for *d*_*HH*_ (Figure 5B), whose value for a neat POPC bilayer equal to 36.7 Å is in good agreement with the literature (Kučerka et al., 2011).

## Discussion

### Bilayer Structure

Our results suggest little changes in the POPC bilayer structure upon the addition of divalent ions Ca^2+^ and Mg^2+^ at their concentrations in the solution up to 50 mM. This may be an unexpected result when considering previously documented changes in the lipid lateral diffusion (Filippov et al., 2009), the tilt of head groups (Seelig, 1990), and ordering of acyl chains (Huster et al., 1999) leading to the alteration of bilayer thickness, hydration, and area per lipid (Uhríková et al., 2008). It is well accepted that divalent ions affect the dynamics and structure of zwitterionic lipid bilayers via their binding to lipid head groups. In particular, Tatulian (1987) reported Ca^2+^- and Mg^2+^-binding constants (*K* = 40 M^−1^ for Ca^2+^ and *K* = 30 M^−1^ for Mg^2+^) for egg phosphatidylcholine—a natural lipid similar chemically to the synthetic POPC, while *K* = 13.8 M^−1^ for Ca^2+^ in POPC at *T* = 25°C (Altenbach and Seelig, 1984). These *K* values turned out to be the same order as the corresponding values for DMPC [*K* = 20 M^−1^ for Ca^2+^ (Gorshkova et al., 2017)] or DPPC [*K* = 19 M^−1^ (Akutsu and Seelig, 1981) or 37 M^−1^ (Satoh, 1995)] bilayers in the fluid phase, where the changes in the structural parameters of a lipid bilayer as a function of the ion concentration were quite pronounced (Uhríková et al., 2007; Uhríková et al., 2008; Kurakin et al., 2021). Despite the difficulties inherent to experimental estimations of the number of adsorbed ions from salt solutions to a lipid bilayer, all reported *K-*values are quite significant in terms of the ability of zwitterionic lipids to bind Ca^2+^. As an example, in the case of POPC (Altenbach and Seelig, 1984), the binding stoichiometry was estimated to be 1 Ca^2+^ ion per 2–3.3 POPC molecules using different evaluation approaches. Similar calculations presented for other phospholipids in articles cited previously conclude typically 1 ion per 1 or 2 lipids. Consequently, the binding stoichiometry of Ca^2+^ ions with a lipid bilayer and the amount of ions adsorbed do not seem to play a primary role in changing its structure.

The absence of changes in *V*_*L*_ (within the error of ∼13 Å^3^) of the POPC MLVs had been reported previously in the Ca^2+^ concentration range of up to 1 M and up to ∼ 300 mM in the case of bilayer thickness, although further there was a significant rigidification of the bilayer (Pabst et al., 2007). In our case, *V*_*L*_ appears to be similarly unaffected by Ca^2+^ and Mg^2+^ in the range of 0–30 mM. In fact, one may expect such a result based on the small sizes of Ca^2+^ and Mg^2+^ cations (a radius of 1 Å and 0.75 Å, respectively). Because of the ion hydration, cations have a weak ability to penetrate deep into the membrane due to high dehydration energy and relatively weak electrostatic effect (compared to the energy of thermal movements of the whole molecule) on uncharged lipid chains, which mainly determine the molecular volume, making significant changes in *V*_*L*_ unlikely.

The absence of changes in the POPC bilayer structure requires yet a different discussion. A possible explanation may be the possibility that ions might crystallize, precipitate, and not dissociate in the solution properly. Several articles devoted to molecular dynamics studies reported the formation of small ionic clusters inhomogeneously distributed in salt solutions even at room temperature (Koneshan and Rasaiah, 2000; Chen and Ruckenstein, 2015). This can potentially lead to a decrease in the ion concentration and in turn, reduce the number of ions bound to the bilayer. However, CaCl_2_ and MgCl_2_ salts are highly soluble at the concentrations utilized in our study. The solubility of calcium and magnesium hydrate complexes in purified water is 56.0 wt% (*T* = 49.4°C) and 36.1 wt% (*T* = 30.0°C), respectively (Clynne and Potter, 1979). Our maximum ion concentration is equal to 50 mM, which corresponds to 0.9 wt% of CaCl_2_⋅2H_2_O and 1.0 wt% of MgCl_2_⋅6H_2_O. In this regard, the ion concentration in our water solution excludes the crystallization or precipitation of ions.

We then turn to the model proposed earlier for describing the bilayer structural changes governed by the area per lipid (Kučerka et al., 2021). This approach is based on the concept of ion bridges that are formed due to the lipid–ion–lipid interactions if the lateral packing density of lipids in the bilayer (related directly to the average interlipid distance < *l* > ∼ $\sqrt{A_{L}}$ ) allows it. For instance, < *l >* of the gel-phase DMPC and DPPC is about 7 Å that is small enough to allow preferential ion bridging, i.e., the neighboring lipids are able to form a local cluster consisting of two or more lipids around a single divalent ion by virtue of electrostatic forces. In contrast, a bilayer composed of DOPC phospholipids has < *l >* larger than 8 Å, which is a cutoff length for the formation of the lipid–ion–lipid bridge (Ronnest et al., 2016). In this case, lipid–ion interactions are preferentially carried out by binding one ion to one lipid. The charge distribution then leads to an increased mutual repulsion of the separated lipid–ion pairs resulting in the expansion of the lateral direction (Kučerka et al., 2021).

**FIGURE 6:**
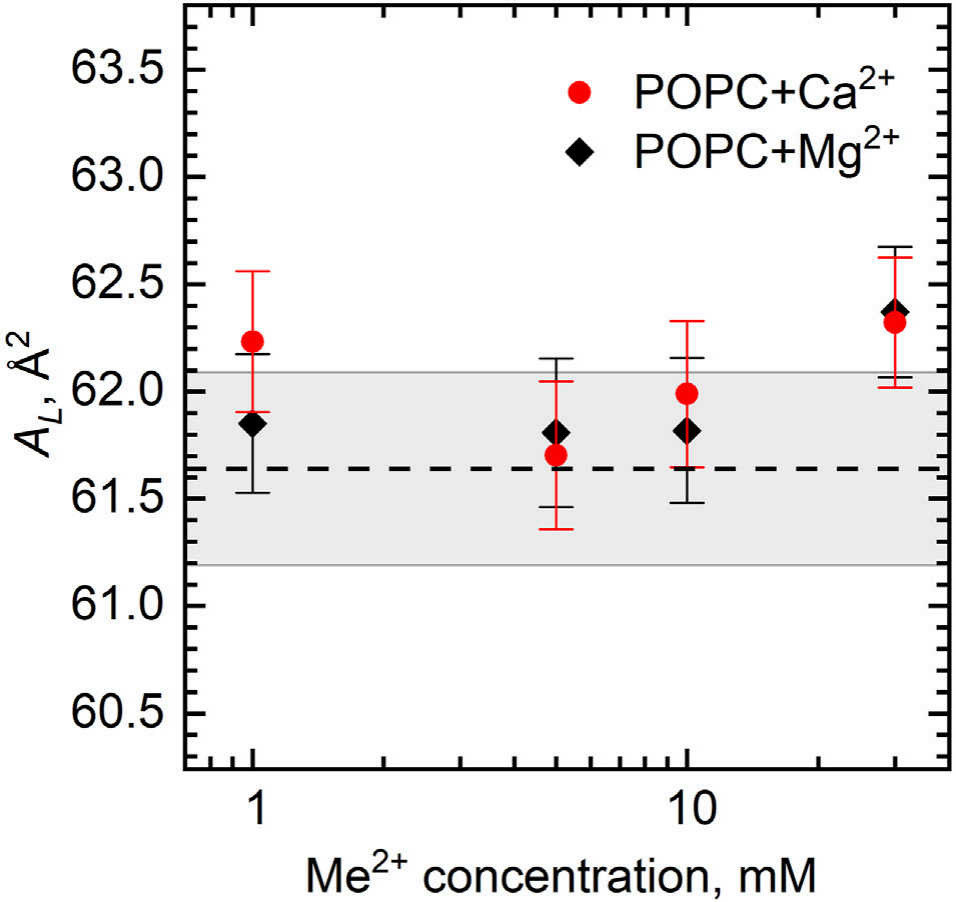
Area per lipid *A*_*L*_ of the POPC bilayer as a function of Ca^2+^ and Mg^2+^ concentrations. The dashed line represents *A*_*L*_ of neat POPC with the wide gray band depicting its standard deviation.

We can calculate *A*_*L*_ directly from our experiments by combining the results of densitometry and SANS as $A_{L}=2V_{L}/d_{L}$ . When adding ions to the POPC bilayer at the concentration range studied, we do not observe the additional strong condensation of lipids leading to the decrease of *A*_*L*_ due to the tight bridging of phospholipids nor the electrostatic repulsion of separated lipid–ion pairs resulting in an increase in *A*_*L*_ (Figure 6). Presumably, this is because POPC has an intermediate area [*A*_*L*_ ∼ 64 Å^2^; note that we have calculated our *A*_*L*_ from SANS data that are known to provide underestimated results (Kučerka et al., 2008)] with an average distance between phospholipids in the bilayer equal to the cutoff length of bridging interactions. Note that our studies have been performed at the fixed temperature (25°C) ensuing the very mentioned area.

The ratio of the number of ion bridges to the number of separated lipid–ion pairs in a lipid bilayer is difficult to calculate precisely. We speculate, based on our experimental results, that the effects of the two interaction modes are presented in the POPC bilayer while compensating each other. Similar to our case, MD simulation results did not show a significant change in *A*_*L*_, although a strong absorption of calcium ions to the POPC bilayer was documented (Melcrová et al., 2016). Other calculations have shown that POPC at a calcium concentration of ∼300 mM forms a 1:1 stoichiometry with a 25% probability and clusters with two or three POPC molecules with almost equal probabilities of ∼35–40% (Melcr et al., 2018). This ratio may, however, depend on the ion concentration, and/or it may relate to the concentration-dependent localization of the Ca^2+^ ion at certain head groups of the POPC (Kučerka et al., 2017a).

### Vesicle Structure

Coming to a discussion of possible changes in the vesicle size, we note that there is some evidence indicating a change in the radius of vesicles in the presence of various salts, monovalent salts, in particular. Yue et al. (2005) have reported an initial increase in the vesicle radius of lipid mixture upon the elevation of ionic strength of the solution being prepared with the sodium ions, whereas Claessens et al. (2004) obtained a strongly nonlinear change in the radius of the DOPG and DOPC vesicles with a pronounced decrease in their size, which was also noticed in the studies of these and other phospholipids (Klasczyk et al., 2010; De Mel et al., 2020).

The divalent metal cations were also found to be an efficient modulator of long-range arrangement in lipid mixtures, particularly for zwitterionic phosphatidylcholines (Inoko et al., 1975; Yamada et al., 2005; Uhríková et al., 2007). Akashi et al. (1998) have reported a decrease of the diameter of giant liposomes from POPC with an increasing CaCl_2_ concentration in the presence of sucrose. The effect apparently depends on the ionic strength that is able to vary bilayer bending moduli resulting from the surface charge density changes in lipid head groups due to the ion absorption onto bilayers. As the charge density is correlated to the average lateral area of lipids (Kučerka et al., 2021), the ion distribution may vary on the vesicle surfaces leading to the changes in the spontaneous curvature. Although some studies (Allolio and Harries, 2021) have reported that Ca^2+^ ions have very little effect on the spontaneous curvature and tilt modulus of POPC vesicles in flat bilayers, others indeed have shown that Ca^2+^ promotes a lipid bilayer to take positive or negative curvature depending on various conditions (Simunovic et al., 2015; Graber et al., 2017). The Ca^2+^ binding to flat and curved bilayers itself has also been reported to differ. Revealed by MD simulations (Magarkar et al., 2017), Ca^2+^ cations collocate primarily with phosphate oxygens in the curved POPC systems corresponding to a vesicle radius of 200 Å. At the same time, Ca^2+^ cations bind to the carbonyl oxygens (although they still preferentially bind to phosphate groups) and interact with the bilayers less readily in the case of planar POPC bilayers.

The additional curvature and different ion binding would lead to the reorganization between lipids in the inner and outer leaflets and emergence of different amounts of lipids between leaflets, most likely already during our extrusion process. Consequently, Ca^2+^ and Mg^2+^ ions may cause lipids to rearrange between two leaflets resulting in the lowest energy of POPC vesicle in the absence of changes in the bilayer structure. A proposed mixed mode of lipid–ion interactions (ion bridges and separated lipid–ion pairs) may be of decisive importance in this case, and they may be involved in this effect of the decrease in size. We note that we have not observed distinct radius changes in our recent studies (Kurakin et al., 2021; Kučerka et al., 2021) performed on the DMPC, DPPC, and DOPC ULVs enriched with Ca^2+^ and Mg^2+^ cations that were concluded to be affected dominantly by lipid–ion–lipid bridging interactions or those of separate lipid–ion pairs, respectively.

It may be worth speculating on a possible effect of the vesicle curvature on the distribution of these interactions between two leaflets of the bilayer. According to Kučerka et al. (2011) and Marquardt et al. (2015), the amount of POPC lipids in the outer leaflet is 1.13 times greater than that in the inner one in the case of a vesicle with 300 Å radius, while this ratio reaches 1.22 in the case of a 200 Å radius vesicle. Although this distribution of the number of lipid molecules evens out the mismatch in the average lipid area, it may not suppress fully the mismatch between the area at the carbonyl groups and the area nearby the P–N dipole. With this in mind, one can estimate the difference between the average area per lipid at the outer/inner leaflets to be 6–8 Å^2^. Intriguingly, then, the difference between the area per lipid at the outer and inner leaflets, balancing around the value of the cutoff length of lipid–ion interactions, might suggest that ion bridges form predominantly at the inner leaflet, while separated ion pairs appear at the outer leaflet. The lipid head groups of the inner leaflet by interacting via ion bridges would then condensate in the lateral direction, while the outer monolayer would be prone to the opposite effect. Mutually, they may indeed change the spontaneous curvature of the bilayer and form smaller vesicles.

## Conclusion

Our small-angle X-ray/neutron scattering and densitometric experimental data have revealed the absence of structural changes (i.e., the bilayer thickness and lateral area per lipid) in the lipid bilayers of POPC unilamellar vesicles enriched with the divalent cations of Ca^2+^ and Mg^2+^ at a fixed T = 25°C. In accordance with our previous results, it is plausible to link these results to a single parameter represented by the area per lipid and thus the interlipid distance. POPC has the area per lipid corresponding to the interlipid distance that coincides with the cutoff length for ion bridging. Hence, the absence of changes might be explained by two different modes of lipid–ion interactions (lipid–ion–lipid bridges and separated lipid–ion pairs) competing with each other. The condensing effect of ion bridges and fluidizing effect of separated lipid–ion pairs on the lipid bilayer are mutually compensated over the entire range of studied ion concentrations.

## Data Availability

The raw data supporting the conclusion of this article will be made available by the authors, without undue reservation.
