# Supplementary material for: Cations Do Not Alter the Membrane Structure of POPC—A Lipid With an Intermediate Area
Source: Front Mol Biosci. 2022 Jul 11;9:926591. doi: 10.3389/fmolb.2022.926591 (PMC9312375; doi:10.3389/fmolb.2022.926591)
Supplement: Supplementary file 1 [file DataSheet1.pdf]

Supplementary Material

1 Supplementary Data

1.1 SANS data analysis

The intensity of scattered neutrons from an isotropic solution of particles is determined by the formula

$$\boldsymbol{I(q) = A\,|F(q)|^2 S(q) + B}, \quad (1)$$

where A is a scaling coefficient depending on the concentration of particles, B is the background intensity, $|F(q)|$ is a form factor of one particle, $S(q)$ is a structural factor that describes the interaction between particles. We have employed the Hayter-Penfold Rescaled Mean Spherical Approximation (RMSA) for the structure factor of charged spheres as available in the SASView fitting software (Hayter and Penfold, 1981; Hansen and Hayter, 1982).

The scattering form factor was calculated using the model of spherical vesicles defined as (Guinier and Fournet, 1955)

$$\boldsymbol{F(q) = \frac{3V_1(\rho_1-\rho_2)j_1(qR_1)}{qR_1} + \frac{3V_2(\rho_2-\rho_1)j_1(qR_2)}{qR_2}}, \quad (2)$$

where V_1 is the volume of the vesicle without the bilayer volume (i.e., solvent filled vesicle core); V_2 is the volume of the vesicle including the bilayer; ρ_1 is the neutron scattering length density (NSLD) of the solution; ρ_2 is the neutron scattering length density of the lipid bilayer; R_1 is the radius of the vesicle core, thus the inner radius of bilayer; R_2 is the outer radius of vesicle; j_1 is the spherical Bessel function of the 1st order.

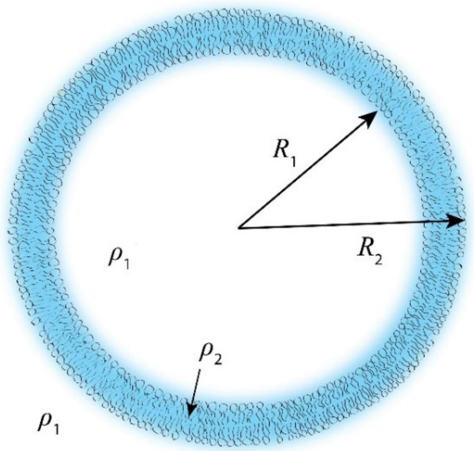

Supplementary Figure S1. Vesicle geometry according to the model of spherical vesicles.

In this model, the scattering intensity is normalized to the volume of a vesicle. The NSLD of the vesicle core is the same as that of the solution outside. In addition, the bilayer-water interface is considered to be sharp since this model does not consider water molecules located in the head groups of phospholipids. This allows for avoiding possible artificial effects that are often present in more complicated multi-parameter models and makes it feasible to track subtle relative changes in the structural parameters of the lipid bilayer and vesicle upon the addition of ions. The employed simple model is justified for SANS data of protonated lipid bilayers dispersed in D_2O due to the abrupt contrast between hydrogen and deuterium containing molecules (Kučerka et al., 2004).

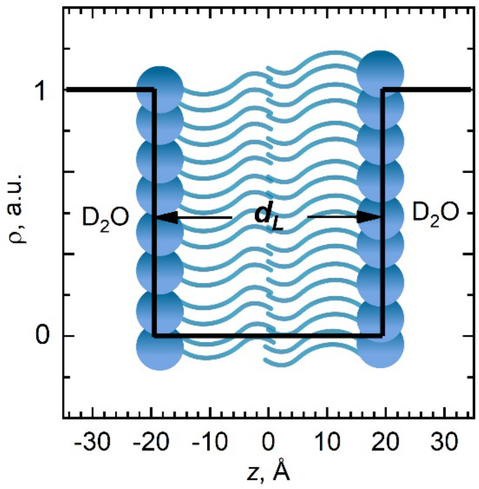

Supplementary Figure S2. Neutron scattering length density distribution of POPC bilayer as used in our SANS data analysis.

The system of extruded lipid vesicles is typical of size polydispersity that we describe by Schulz distribution function (Helfrich, 1986; Hallett et al., 1991)

$$f(R_1) = \frac{1}{R_0}(z+1)^{z+1}\frac{1}{G(z+1)}\left(\frac{R_1}{R_0}\right)^z \exp\left(-\frac{(z+1)R_1}{R_0}\right), \quad (4)$$

where

$$z = \left(\frac{R_0}{\sigma_R}\right)^2 - 1, \quad (5)$$

G(z) is the gamma function ($z > 0$), R_0 is the median value of this distribution. The form factor of the polydisperse vesicles is then obtained by convoluting the single-particle function with the given distribution function.

1.2 SAXS data analysis

SAXS curves were fitted following the factorization of form factor obtained for a large particle with a thin shell (Porod, 1948). Therefore, the intensity may be written as

$$I(q) = A\,P_{sh}(q)\,|F(q)|^2 + B, \quad (6)$$

where A is a scaling factor, $P_{sh}(q)$ is the form-factor of the thin spherical shell with radius R (vesicle), and $F(q)$ is the form-factor of the lipid bilayer. $P_{sh}(q)$ is defined as (Porod, 1948)

$$\boldsymbol{P_{sh}(q) = \int_0^{\infty} f(R',\sigma,R)\left(\frac{4\pi R'^2 \sin(qR')}{qR'}\right)^2 dR'}, \tag{7}$$

where $f(R',\sigma,R)$ is an asymmetric log-normal distribution describing the vesicle polydispersity as

$$\boldsymbol{f(R',\sigma,R) = \frac{1}{\sqrt{2\pi}}\frac{1}{R\sigma}\exp\left\{-\frac{1}{2}\left(\frac{\ln R-\mu}{\sigma}\right)^2\right\}}, \tag{8}$$

with $\mu = \ln R_m$ and $\sigma = \frac{p}{R_m}$, where R_m is the median value and p is the deviation from the median value. The mean size value is then $R_{mean} = \exp(\mu + \sigma^2/2)$. Log-normal distribution has proved to fit well the small-angle X-ray and neutron scattering data related to the large particles and vesicles in particular, as well as the dynamic light scattering data, while revealing the similar results when compared to other distributions (Pencer et al., 2001).

Given the symmetric lipid bilayer, $F(q)$ is calculated as the Fourier transform of the electron density profile of bilayer

$$\boldsymbol{F(q) = \int_{-\frac{d}{2}}^{+\frac{d}{2}} \rho(z)\cos(qz)\,dz}, \tag{9}$$

We describe $\rho(z)$ by 3 Gauss functions according to well established approach (Pabst et al., 2000)

$$\boldsymbol{\rho(z) = \rho_{head}\left[\exp\left(-\frac{\left(z-\frac{z_{head}}{2}\right)^2}{2\sigma_{head}^2}\right) + \exp\left(-\frac{\left(z+\frac{z_{head}}{2}\right)^2}{2\sigma_{head}^2}\right)\right] + \rho_{tail}\left(-\frac{z^2}{2\sigma_{tail}^2}\right)}, \tag{10}$$

where ρ_{head} and ρ_{tail} are electron densities of lipid head groups and tails relative to the water electron density. Positions of the Gaussian peaks are at z_i with the widths of σ_i. Hence, the form factor is given by

$$\boldsymbol{F(q) = 2\sqrt{2\pi}\sigma_{head}\rho_{head}\exp\left(-\frac{1}{2}(q\sigma_{head})^2\right) + \sqrt{2\pi}\sigma_{tail}\rho_{tail}\exp\left(-\frac{1}{2}(q\sigma_{tail})^2\right)}, \tag{11}$$

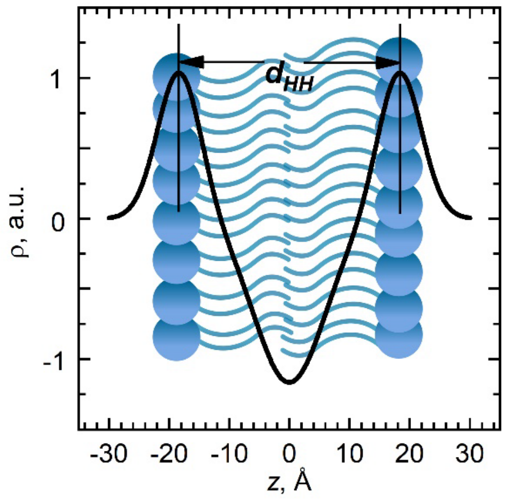

Supplementary Figure S3. Electron density distribution of POPC bilayer as used in our SAXS data analysis.

1.3 Densitometry

The specific volume of lipid v_L was determined from densitometric measurements according to the formula (Pabst et al., 2007)

$$\boldsymbol{v_L = \frac{v_s - (1 - w_L)v_{buffer}}{w_L}}, \tag{12}$$

where v_s is the specific volume that is inverse to the measured density of the lipid dispersion at a specific ion concentration, v_{buffer} is the specific volume of the corresponding buffer that has been used for the preparation of lipid dispersions, w_L is the mass fraction of lipid in the sample.

Considering the specific volume, the average molecular volume of lipids may be calculated as

$$\boldsymbol{V_L = \frac{v_L M}{N_A}}, \tag{13}$$

where N_A is the Avogadro constant, M is the molar mass of POPC.

2 Supplementary References

Guinier, A., and Fournet, G. (1955). *Small-Angle Scattering of X-Rays.* John Wiley and Sons, New York.

Hallett, F.R., Watton, J., and Krygsman, P. (1991). Vesicle sizing: Number distributions by dynamic light scattering. *Biophys J* 59(2)**,** 357-362. doi: 10.1016/S0006-3495(91)82229-9.

Hansen, J.-P., and Hayter, J.B. (1982). A rescaled MSA structure factor for dilute charged colloidal dispersions. *Molecular Physics* 46(3)**,** 651-656. doi: 10.1080/00268978200101471.

Hayter, J.B., and Penfold, J. (1981). An analytic structure factor for macroion solutions. *Molecular Physics* 42(1)**,** 109-118. doi: 10.1080/00268978100100091.

Helfrich, W. (1986). Size distributions of vesicles : the role of the effective rigidity of membranes. *J. Phys. France* 47(2)**,** 321-329. doi: 10.1051/jphys:019860047020321 00.

Kučerka, N., Nagle, J.F., Feller, S.E., and Balgavý, P. (2004). Models to analyze small-angle neutron scattering from unilamellar lipid vesicles. *Physical Review E* 69(5), 051903. doi: 10.1103/PhysRevE.69.051903.

Pabst, G., Hodzic, A., Štrancar, J., Danner, S., Rappolt, M., and Laggner, P. (2007). Rigidification of Neutral Lipid Bilayers in the Presence of Salts. *Biophysical Journal* 93(8), 2688-2696. doi: 10.1529/biophysj.107.112615.

Pabst, G., Rappolt, M., Amenitsch, H., and Laggner, P. (2000). Structural information from multilamellar liposomes at full hydration: Full q-range fitting with high quality x-ray data. *Physical Review E* 62(3), 4000-4009. doi: 10.1103/PhysRevE.62.4000.

Pencer, J., White, G.F., and Hallett, F.R. (2001). Osmotically Induced Shape Changes of Large Unilamellar Vesicles Measured by Dynamic Light Scattering. *Biophysical Journal* 81(5), 2716-2728. doi: 10.1016/S0006-3495(01)75914-0.

Porod, G. (1948). *Acta Phys. Austriaca* 2, 255-292.
